# Factors influencing the efficacy and safety of esaxerenone in hypertensive patients: a pooled analysis of five clinical studies on different comorbidities

**DOI:** 10.1038/s41440-024-01818-0

**Published:** 2024-08-02

**Authors:** Kazuomi Kario, Tomohiro Katsuya, Jun Wada, Hirohiko Motoki, Koichiro Kuwahara, Kenichi Tsujita, Takashi Taguchi, Ayumi Tanabe, Tatsuo Shimosawa

**Affiliations:** 1 Division of Cardiovascular Medicine, Department of Medicine, Jichi Medical University School of Medicine, Tochigi, Japan; 2 Katsuya Clinic, Hyogo, Japan; 3 Department of Nephrology, Rheumatology, Endocrinology and Metabolism, Okayama University Faculty of Medicine, Dentistry and Pharmaceutical Sciences, Okayama, Japan; 4 Department of Cardiovascular Medicine, Shinshu University School of Medicine, Nagano, Japan; 5 Department of Cardiovascular Medicine, Graduate School of Medical Sciences, Kumamoto University, Kumamoto, Japan; 6 Primary Medical Science Department, Daiichi Sankyo Co. Ltd., Tokyo, Japan; 7 Data Intelligence Department, Daiichi Sankyo Co. Ltd., Tokyo, Japan; 8 Department of Clinical Laboratory, School of Medicine, International University of Health and Welfare, Chiba, Japan

**Keywords:** Antihypertensive, Clinical study, Esaxerenone, Hyperkalemia, Pooled analysis

## Abstract

This study aimed to identify factors associated with a strong home blood pressure (BP)-lowering effect of esaxerenone and the incidence of elevated serum potassium levels in hypertensive patients treated with esaxerenone. A pooled analysis of five multicenter, prospective, open-label single-arm studies was conducted, including 479 patients in the full analysis set (FAS) and 492 patients in the safety analysis set. Multivariate linear regression analysis of morning home systolic BP (SBP) and diastolic BP (DBP) changes from baseline to Week 12 in the FAS (primary endpoint) showed that male sex (estimated change 4.37 mmHg), office pulse rate ≥100 beats/min (25.10 mmHg), and calcium channel blocker (CCB) use as a basal antihypertensive agent (4.53 mmHg) were significantly associated with a positive estimated change (weaker BP-lowering effect) in morning home SBP. CCB use (3.70 mmHg) was associated with a positive estimated change in morning home DBP. Urine albumin‐to‐creatinine ratio 30 to <300 mg/gCr (−4.13 mmHg) was significantly associated with a negative estimated change (stronger BP-lowering effect) in morning home SBP. Based on multivariate logistic regression analysis, elevated baseline serum potassium level (≥4.5 vs < 4.5 mEq/L, odds ratio 13.502) was significantly associated with a high incidence of serum potassium level ≥5.5 mEq/L after esaxerenone treatment. In conclusion, factors associated with a strong BP-lowering effect of esaxerenone were female sex and use of renin–angiotensin system inhibitors as a basal antihypertensive drug. Patients with baseline serum potassium levels ≥4.5 mEq/L had an increased risk of developing elevated serum potassium levels (≥5.5 mEq/L) after esaxerenone treatment.

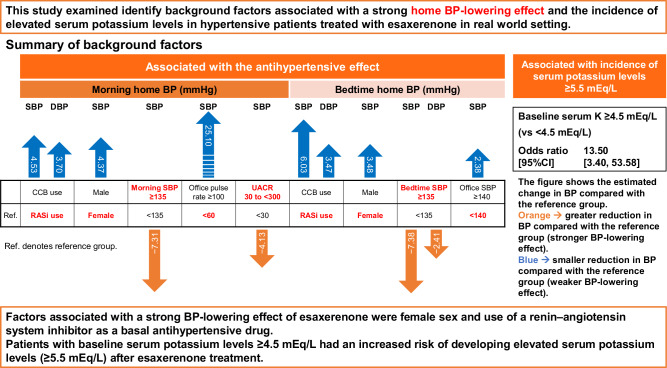

## Introduction

Hypertension is a known risk factor for cerebrovascular and cardiovascular events. Home blood pressure (BP) and ambulatory BP monitoring have been shown to have a stronger association with poor cardiovascular prognosis compared with office BP [1–5]. Morning home BP has a particularly strong association with cardiovascular events and progression of diabetic nephropathy [6–16]. Currently, the 2019 Japanese Society of Hypertension Guidelines recommend the use of home BP measurements for the diagnosis of hypertension and to evaluate the efficacy of antihypertensive medications [17]. However, the BP-lowering effect of antihypertensive agents is limited, and achieving adequate control of morning home BP is difficult in many patients [18, 19].

Esaxerenone, a next-generation non-steroidal mineralocorticoid receptor blocker (MRB), exhibits greater selectivity, potency, and longer half-life compared with other MRBs, and has favorable antihypertensive effects [20]. In clinical settings, esaxerenone has been evaluated in hypertensive patients with diverse underlying medical conditions, including diabetic kidney disease, patients with diabetes mellitus who are treated with sodium-glucose cotransporter 2 inhibitors, left ventricular hypertrophy, nocturnal hypertension, and patients with inadequate antihypertensive response to angiotensin receptor blockers or calcium channel blockers (CCBs). These studies have reported on the antihypertensive effect of esaxerenone (office and home BP) and its organ-protective effects, such as reducing urine albumin‐to‐creatinine ratio (UACR) and N-terminal pro-brain natriuretic peptide (NT-proBNP) levels [21–25]. Additionally, previous studies in hypertensive patients treated with esaxerenone have also shown a significant improvement in nocturnal BP, morning and bedtime home BP, and office BP, significant changes from riser/non-dipper patterns to dipper/extreme dipper patterns, and a sustained 24 h BP-lowering effect [23, 26–28].

A previous pooled analysis of seven phase 3 clinical trials investigated background factors associated with the antihypertensive effect of esaxerenone [29]. In that study, the office BP-lowering effect of esaxerenone after 12 weeks of treatment was found to be associated with female sex, low body weight, low plasma aldosterone concentration, short duration of hypertension, no history of antihypertensive medication, low UACR, and non-smoker status [29]. Additionally, the study investigated factors associated with an increase in serum potassium levels during esaxerenone treatment [29]. Elevated serum potassium levels are a well-known class adverse effect of esaxerenone [30]. High serum potassium, low estimated glomerular filtration rate (eGFR), and high UACR were associated with an increase in serum potassium levels with esaxerenone administration [29], which are already included in the package insert [31]. However, there is a lack of studies evaluating factors associated with the home BP-lowering effect of esaxerenone, as well as other potential factors related to safety concerns of serum potassium elevation in real clinical conditions.

We hypothesized that there is a subgroup of hypertensive patients who can significantly improve home BP control with esaxerenone treatment in real clinical conditions. By evaluating a hypertensive population with background factors more similar to real clinical conditions, we may identify patients who could benefit more from esaxerenone treatment within real-world settings.

This study aimed to identify background factors associated with a strong home BP-lowering effect of esaxerenone and the incidence of elevated serum potassium levels in hypertensive patients treated with esaxerenone.

## Methods

### Study design and patients

This pooled analysis included five clinical studies on esaxerenone: EX-DKD [21], EARLY-NH [23], ESES-LVH [24], ENaK [25], and EAGLE-DH [22]. These studies were multicenter, prospective, open-label single-arm studies. The target populations of each study are described in Table 1. In the studies, patients received esaxerenone along with basal antihypertensive medications such as angiotensin receptor blockers, CCBs, or renin–angiotensin system (RAS) inhibitors.

**Table 1:** Changes in home and office BP and frequency of elevated serum potassium level in each study

Study	Target population	N patients in FAS	Mean change from baseline in SBP/DBP, mmHg									Frequency of serum potassium elevation ≥ 5.5 mEq/L, n (%)
			Morning home BP			Bedtime home BP			Office BP			
			BP at baseline	Week 12	EOT^a^	BP at baseline	Week 12	EOT^a^	BP at baseline	Week 12	EOT^a^	
EX-DKD	Hypertensive patients with diabetic kidney disease	109	135.6/75.9	−12.0/ − 5.2	−11.6/ − 5.2	129.3/71.0	−10.9/ − 4.8	−10.0/ − 4.4	144.7/76.1	−12.7/ − 5.4	−11.5/ − 5.2	3 (2.7)
EARLY-NH	Patients with nocturnal hypertension	93	143.8/86.7	−11.9/ − 4.2	−12.2/ − 4.9	135.1/80.5	−10.6/ − 4.2	−10.8/ − 4.2	147.2/84.1	−10.4/ − 4.9	−11.1/ − 5.5	11 (10.9)
ESES-LVH	Hypertensive patients with left ventricular hypertrophy	58	142.8/85.0	−10.5/ − 4.8	−11.9/ − 5.0	141.0/81.9	−11.1/ − 5.4	−11.8/ − 4.6	145.9/85.3	−13.9/ − 5.9	−14.9/ − 7.3	3 (5.0)
ENaK	Patients with essential hypertension	126	136.7/88.0	−12.3/ − 6.7	−11.9/ − 6.4	130.5/82.0	−10.6/ − 6.0	−10.7/ − 5.7	141.4/86.5	−12.5/ − 7.9	−12.3/ − 7.9	9 (7.1)
EAGLE-DH	Hypertensive patients with diabetes taking SGLT2 inhibitors	93	136.4/82.3	−11.8/ − 5.1	−12.9/ − 5.7	131.9/78.0	−10.6/ − 4.8	−11.8/ − 5.5	136.5/80.0	−11.3/ − 6.8	−11.8/ − 7.1	1 (1.1)

*BP* blood pressure, *DBP* diastolic blood pressure, *EOT* end of treatment, *FAS* full analysis set, *SBP* systolic blood pressure, *SGLT2* sodium-glucose cotransporter-2

^a^The dosing period for ESES-LVH and EAGLE-DH was 24 weeks

Ethical approval for this study was obtained from the ethical review committee of the Kitamachi Clinic (Tokyo, Japan), and the study was conducted in accordance with the ethical principles outlined in the Declaration of Helsinki and local laws and regulations. The requirement for informed consent was waived because the present study involved secondary use of data from five previous studies. The five studies were registered at the Japan Registry of Clinical Trials (jRCT) under the following identifier numbers: jRCTs061190027 (EX-DKD), jRCTs031200364 (EARLY-NH), jRCTs071190043 (ESES-LVH), jRCTs031210273 (ENaK), and jRCTs031200273 (EAGLE-DH). The current study is registered at the University Hospital Medical Information Network Clinical Trials Registry (UMIN): UMIN000051525.

### Endpoints

The primary endpoint was to evaluate which background factors were associated with changes in morning home systolic BP (SBP) and diastolic BP (DBP) from baseline to Week 12. The secondary endpoint was to evaluate which background factors were associated with changes in bedtime home and office BP from baseline to Week 12. The safety endpoint was to evaluate which background factors were associated with a high incidence of elevated serum potassium levels (≥5.5 mEq/L) up to Week 12.

### Statistical methods

The primary and secondary endpoints were evaluated in the full analysis set (FAS) of each study, while the safety endpoints were evaluated in the safety analysis set (SAS) of each study. For the primary and secondary endpoints, multivariate linear regression models were used to evaluate the effect of each background factor on the change in BP, and 95% confidence intervals (CIs) and *P* values were calculated using the changes in morning home BP, bedtime home BP, and office BP (SBP and DBP) from baseline to Week 12 as objective variables. For the safety endpoint, odds ratios (ORs), their 95% CIs, and *P* values for each background factor were calculated using multivariate logistic regression models, with the onset of serum potassium elevation (≥5.5 mEq/L) up to Week 12 as an outcome. In all models, the dosing regimen was used as a common covariate. Missing BP measurements after the start of study drug administration were imputed using the last observation carried forward method. All statistical analyses were performed using SAS software version 9.4 (SAS Institute Inc., Cary, NC, USA).

## Results

### Patients

The number of patients in the FAS and SAS, respectively, of each study was as follows: EX-DKD (n = 109 and 112), EARLY-NH (n = 93 and 101), ESES-LVH (n = 58 and 60), ENaK (both n = 126), and EAGLE-DH (both n = 93). Therefore, a total of 479 were included in the analysis for the primary and secondary endpoints, and 492 were included in the analysis of the safety endpoint.

The changes in morning and bedtime home BP and office BP from baseline to Week 12 and end of treatment, as well as the frequency of elevated serum potassium levels ≥5.5 mEq/L in each study, are summarized in Table 1. In all five studies, significant reductions in morning home BP, bedtime home BP, and office BP from baseline to Week 12 or end of treatment were observed in patients treated with esaxerenone. The proportion of patients with serum potassium levels ≥5.5 mEq/L ranged from 1.1% to 10.9%.

Baseline demographic and clinical characteristics of patients in the FAS and SAS are summarized in Table 2. In the FAS, 57.8% of patients were male and the mean ± SD age was 66.5 ± 11.3 years; body mass index, 25.9 ± 4.2 kg/m^2^; morning home SBP/DBP, 138.4/83.5 ± 12.1/10.5 mmHg; creatinine-based eGFR (eGFR_creat_), 66.0 ± 18.1 mL/min/1.73 m^2^; and serum potassium level, 4.2 ± 0.4 mEq/L. The proportion of patients with diabetes was 58.2%, and that of patients with prior use of basal antihypertensive drugs was 41.3% for RAS inhibitors, 34.2% for CCBs, and 24.4% for both drugs. Similar patient baseline characteristics were observed in the SAS. Baseline demographic and clinical characteristics of patients in each of the five clinical studies are summarized in Supplementary Table 1.

**Table 2:** Baseline demographic and clinical characteristics

	FAS n = 479	SAS n = 492
Sex, n (%)		
Male	277 (57.8)	284 (57.7)
Female	202 (42.2)	208 (42.3)
Age, years		
N	479	492
Mean ± SD	66.5 ± 11.3	66.7 ± 11.3
Median (range)	69.0 (27, 92)	69.0 (27, 92)
BMI, kg/m^2^		
N	478	490
Mean ± SD	25.9 ± 4.2	25.9 ± 4.2
Complications, n (%)		
Any	440 (92.4)	452 (92.4)
Diabetes	279 (58.2)	283 (57.5)
Dyslipidemia	285 (68.0)	294 (68.4)
Hyperuricemia	94 (22.4)	95 (22.1)
Morning home SBP, mmHg		
N	472	485
Mean ± SD	138.4 ± 12.1	138.5 ± 12.2
Morning home DBP, mmHg		
N	472	485
Mean ± SD	83.5 ± 10.5	83.4 ± 10.5
Bedtime home SBP, mmHg		
N	472	484
Mean ± SD	132.6 ± 13.3	132.6 ± 13.3
Bedtime home DBP, mmHg		
N	472	484
Mean ± SD	78.4 ± 10.8	78.4 ± 10.9
Office SBP, mmHg		
N	479	492
Mean ± SD	142.9 ± 15.3	142.9 ± 15.4
Office DBP, mmHg		
N	479	492
Mean ± SD	82.3 ± 11.7	82.2 ± 11.7
Morning home pulse rate, beats/min		
N	459	472
Mean ± SD	68.5 ± 9.7	68.5 ± 9.8
Bedtime home pulse rate, beats/min		
N	457	469
Mean ± SD	72.5 ± 10.5	72.5 ± 10.5
Office pulse rate, beats/min		
N	479	492
Mean ± SD	73.3 ± 11.3	73.2 ± 11.4
Duration of hypertension, years		
N	335	347
Mean ± SD	9.4 ± 8.5	9.5 ± 8.7
Basal antihypertensive, n (%)		
RAS inhibitor	198 (41.3)	204 (41.5)
CCB	164 (34.2)	169 (34.3)
RAS inhibitor/CCB	117 (24.4)	119 (24.2)
Serum potassium, mEq/L		
N	469	482
Mean ± SD	4.2 ± 0.4	4.2 ± 0.4
eGFR_creat_, mL/min/1.73 m^2^		
N	469	482
Mean ± SD	66.0 ± 18.1	65.8 ± 18.0
UACR, mg/gCr		
N	476	489
Mean ± SD	110.4 ± 349.4	109.6 ± 345.7
NT-proBNP, pg/mL		
N	463	476
Mean ± SD	103.0 ± 152.2	106.3 ± 162.6
Plasma aldosterone concentration, pg/mL		
N	443	456
Mean ± SD	49.1 ± 35.6	50.3 ± 46.5
Plasma renin activity, ng/mL/h		
N	470	483
Mean ± SD	3.6 ± 9.5	3.5 ± 9.4
Smoking habit, n (%)		
No	396 (82.7)	408 (82.9)
Yes	83 (17.3)	84 (17.1)
Drinking habit, n (%)		
No	267 (56.1)	275 (56.2)
Yes	209 (43.9)	214 (43.8)
Urinary sodium, mEq/L		
N	479	492
Mean ± SD	108.5 ± 50.8	108.5 ± 50.6
Urinary potassium, mEq/L		
N	478	491
Mean ± SD	40.4 ± 23.4	40.4 ± 23.4
Initial esaxerenone dose, n (%)		
1.25 mg	230 (48.0)	236 (48.0)
2.5 mg	249 (52.0)	256 (52.0)

*BMI* body mass index, *CCB* calcium channel blocker, *DBP* diastolic blood pressure, *eGFR*_*creat*_ creatinine-based estimated glomerular filtration rate, *FAS* full analysis set, *NT-proBNP* N-terminal pro-brain natriuretic peptide, *RAS* renin–angiotensin system, *SAS* safety analysis set, *SBP* systolic blood pressure, *SD* standard deviation, *UACR* urine albumin‐to‐creatinine ratio

### Background factors associated with the morning BP-lowering effect of esaxerenone

Table 3 shows the results of linear regression analysis of morning home SBP and DBP changes from baseline to Week 12 in the FAS (primary endpoint). The following factors were significantly associated with a positive estimated change (weaker BP-lowering effect) in morning home SBP from baseline to Week 12 after esaxerenone treatment compared with the reference group: male sex (estimated change 4.37 mmHg; *P* = 0.031), office pulse rate ≥100 beats/min (estimated change 25.10 mmHg; *P* = 0.044), and CCB use as a basal antihypertensive agent (estimated change 4.53 mmHg; *P* = 0.042). UACR 30 to <300 mg/gCr (estimated change −4.13 mmHg; *P* = 0.043) was significantly associated with a negative estimated change (stronger BP-lowering effect) in morning home SBP from baseline to Week 12 after esaxerenone treatment. CCB use as a basal antihypertensive agent (estimated change 3.70 mmHg; *P* = 0.015) was associated with a positive estimated change in morning home DBP from baseline to Week 12 after esaxerenone treatment.

**Table 3:** Multivariate analysis of factors associated with a change from baseline to Week 12 in morning home SBP and DBP in the FAS (n = 479)

	N	SBP			DBP		
		Estimated change in BP, mmHg	95% CI	*P*	Estimated change in BP, mmHg	95% CI	*P*
Sex							
Male	277	4.37	0.40, 8.35	0.031	1.71	−1.01, 4.43	0.218
Female	202	Ref			Ref		
Age, years							
<65	175	Ref			Ref		
65 to <75	185	−0.53	−4.77, 3.71	0.805	−1.29	−4.19, 1.61	0.380
≥75	119	−1.98	−7.22, 3.25	0.456	−1.12	−4.70, 2.46	0.537
BMI, kg/m^2^							
<18.5	8	−0.04	−22.49, 22.41	0.997	2.23	−13.12, 17.59	0.775
18.5 to <25	207	Ref			Ref		
≥25	263	0.85	−2.65, 4.36	0.631	−0.42	−2.81, 1.98	0.731
Morning home SBP, mmHg							
<135	187	Ref			Ref		
≥135	285	−7.31	−11.42, −3.21	<0.001	−1.97	−4.78, 0.84	0.168
Morning home DBP, mmHg							
<85	256	Ref			Ref		
≥85	216	−0.88	−5.62, 3.86	0.715	−2.65	−5.90, 0.59	0.108
Bedtime home SBP, mmHg							
<135	268	Ref			Ref		
≥135	204	−0.17	−4.23, 3.88	0.932	−0.48	−3.25, 2.29	0.731
Bedtime home DBP, mmHg							
<85	346	Ref			Ref		
≥85	126	1.59	−3.48, 6.67	0.536	0.53	−2.95, 4.00	0.765
Office SBP, mmHg							
<140	204	Ref			Ref		
≥140	275	0.55	−3.26, 4.36	0.777	0.40	−2.20, 3.01	0.761
Office DBP, mmHg							
<90	351	Ref			Ref		
≥90	128	2.68	−1.98, 7.35	0.258	1.85	−1.34, 5.05	0.253
Morning home pulse rate, beats/min							
<60	73	Ref			Ref		
60 to <100	384	−2.28	−9.07, 4.52	0.510	−2.30	−6.94, 2.35	0.331
≥100	2	–			–		
Bedtime home pulse rate, beats/min							
<60	48	Ref			Ref		
60 to <100	404	−2.26	−10.06, 5.54	0.568	0.49	−4.84, 5.82	0.856
≥100	5	–			–		
Office pulse rate, beats/min							
<60	35	Ref			Ref		
60 to <100	435	6.57	−2.49, 15.64	0.154	−0.11	−6.31, 6.09	0.972
≥100	9	25.10	0.65, 49.54	0.044	8.22	−8.50, 24.94	0.333
Duration of hypertension, years							
<5	126	Ref			Ref		
5 to <10	80	1.50	−2.81, 5.82	0.493	1.63	−1.32, 4.58	0.278
≥10	129	0.60	−3.46, 4.66	0.771	0.92	−1.85, 3.70	0.513
Basal antihypertensive drugs							
RAS inhibitor	198	Ref			Ref		
CCB	164	4.53	0.17, 8.89	0.042	3.70	0.72, 6.68	0.015
RAS inhibitor/CCB	117	2.41	−1.89, 6.72	0.270	2.05	−0.89, 4.99	0.171
Serum potassium, mEq/L							
<4.5	353	Ref			Ref		
≥4.5	116	−0.88	−5.02, 3.26	0.675	−0.30	−3.13, 2.53	0.834
eGFR_creat_, mL/min/1.73 m^2^							
30 to <60	213	NE			NE		
≥60	256	Ref			Ref		
UACR, mg/gCr							
<30	301	Ref			Ref		
30 to <300	129	−4.13	−8.13, −0.13	0.043	−1.39	−4.12, 1.34	0.317
≥300	46	−3.60	−10.03, 2.83	0.270	−1.17	−5.57, 3.23	0.600
NT-proBNP, pg/mL							
<125	371	Ref			Ref		
125 to <400	73	2.14	−2.64, 6.93	0.378	1.10	−2.17, 4.37	0.507
≥400	19	−2.43	−13.00, 8.13	0.650	−3.34	−10.56, 3.88	0.363
Plasma aldosterone concentration, pg/mL							
<120	426	Ref			Ref		
≥120	17	−0.53	−10.31, 9.25	0.915	−2.69	−9.38, 4.00	0.428
Plasma renin activity, ng/mL/h							
<1.0	183	Ref			Ref		
≥1.0	287	−0.12	−3.73, 3.49	0.947	−0.15	−2.61, 2.32	0.907
Complications							
No	36	Ref			Ref		
Yes	440	–			–		
Diabetes							
No	200	Ref			Ref		
Yes	279	−0.27	−4.95, 4.40	0.908	0.21	−2.98, 3.41	0.895
Dyslipidemia							
No	134	Ref			Ref		
Yes	285	1.78	−2.04, 5.60	0.359	0.86	−1.75, 3.47	0.517
Hyperuricemia							
No	325	Ref			Ref		
Yes	94	0.73	−3.49, 4.95	0.732	0.11	−2.77, 3.00	0.938
Smoking habit							
No	396	Ref			Ref		
Yes	83	2.20	−2.82, 7.22	0.388	2.05	−1.38, 5.48	0.240
Drinking habit							
No	267	Ref			Ref		
Yes	209	2.57	−1.09, 6.24	0.167	0.78	−1.72, 3.29	0.537
Initial dose of esaxerenone, mg							
1.25	230	Ref			Ref		
2.5	249	−3.49	−7.36. 0.39	0.078	−1.68	−4.33, 0.97	0.212

Estimates (95% CIs) indicate the absolute difference in BP change compared with the reference category. Negative estimates indicate a greater decrease in BP relative to the reference (i.e., a relatively stronger antihypertensive effect), whereas positive estimates indicate a smaller decrease in BP relative to the reference (i.e., a relatively weaker antihypertensive effect)

NE denotes variables excluded because the variance inflation factor was >5.0

*BMI* body mass index, *BP* blood pressure, *CCB* calcium channel blocker, *CI* confidence interval, *DBP* diastolic blood pressure, *eGFR*_*creat*_ creatinine-based estimated glomerular filtration rate, *FAS* full analysis set, *NE* not examined, *NT-proBNP* N-terminal pro-brain natriuretic peptide, *RAS* renin–angiotensin system, *Ref* reference, *SBP* systolic blood pressure, *UACR* urine albumin‐to‐creatinine ratio

### Background factors associated with the bedtime home and office BP-lowering effect of esaxerenone

Table 4 shows the results of linear regression analysis of bedtime home SBP and DBP changes from baseline to Week 12 in the FAS (secondary endpoint). The following factors were significantly associated with a positive estimated change in bedtime home SBP from baseline to Week 12 after esaxerenone treatment compared with the reference group: male sex (estimated change 3.48 mmHg; *P* = 0.045) and CCB use (estimated change 6.03 mmHg; *P* = 0.002).

**Table 4:** Multivariate analysis of factors associated with a change from baseline to Week 12 in bedtime home SBP and DBP in the FAS (n = 479)

	N	SBP			DBP		
		Estimated change in BP, mmHg	95% CI	*P*	Estimated change in BP, mmHg	95% CI	*P*
Sex							
Male	277	3.48	0.08, 6.88	0.045	0.83	−1.18, 2.85	0.417
Female	202	Ref			Ref		
Age, years							
<65	175	Ref			Ref		
65 to <75	185	0.82	−2.78, 4.42	0.654	−0.02	−2.16, 2.12	0.987
≥75	119	−1.39	−5.85, 3.07	0.539	−1.21	−3.86, 1.44	0.368
BMI, kg/m^2^							
<18.5	8	2.49	−16.67, 21.65	0.798	9.47	−1.90, 20.84	0.102
18.5 to <25	207	Ref			Ref		
≥25	263	1.45	−1.53, 4.44	0.338	0.15	−1.62, 1.93	0.865
Morning home SBP, mmHg							
<135	187	Ref			Ref		
≥135	285	−1.88	−5.37, 1.62	0.291	−0.73	−2.81, 1.34	0.488
Morning home DBP, mmHg							
<85	256	Ref			Ref		
≥85	216	1.57	−2.46, 5.61	0.443	−0.56	−2.95, 1.84	0.648
Bedtime home SBP, mmHg							
<135	268	Ref			Ref		
≥135	204	−7.38	−10.77, −3.98	<0.001	−2.41	−4.42, −0.39	0.020
Bedtime home DBP, mmHg							
<85	346	Ref			Ref		
≥85	126	−0.94	−5.28, 3.40	0.669	−1.60	−4.18, 0.97	0.221
Office SBP, mmHg							
<140	204	Ref			Ref		
≥140	275	2.76	−0.45, 5.98	0.092	2.38	0.47, 4.29	0.015
Office DBP, mmHg							
<90	351	Ref			Ref		
≥90	128	1.68	−2.27, 5.63	0.401	0.58	−1.76, 2.93	0.625
Morning home pulse rate, beats/min							
<60	73	Ref			Ref		
60 to <100	384	2.69	−3.13, 8.50	0.363	−0.34	−3.79, 3.11	0.847
≥100	2	–			–		
Bedtime home pulse rate, beats/min							
<60	48	Ref			Ref		
60 to <100	404	−5.79	−12.45, 0.87	0.088	−1.45	−5.40, 2.50	0.470
≥100	5	–			–		
Office pulse rate, beats/min							
<60	35	Ref			Ref		
60 to <100	435	3.83	−3.91, 11.56	0.330	−1.23	−5.82, 3.36	0.597
≥100	9	13.38	−2.83, 29.59	0.105	−3.51	−13.13, 6.11	0.472
Duration of hypertension, years							
<5	126	Ref			Ref		
5 to <10	80	1.07	−2.61, 4.75	0.567	2.16	−0.03, 4.34	0.053
≥10	129	−0.35	−3.81, 3.11	0.841	1.51	−0.55, 3.56	0.150
Basal antihypertensive drugs							
RAS inhibitor	198	Ref			Ref		
CCB	164	6.03	2.34, 9.72	0.002	3.47	1.29, 5.66	0.002
RAS inhibitor/CCB	117	2.32	−1.30, 5.95	0.208	1.55	−0.60, 3.70	0.157
Serum potassium, mEq/L							
<4.5	353	Ref			Ref		
≥4.5	116	0.98	−2.54, 4.49	0.584	1.01	−1.08, 3.09	0.343
eGFR_creat_, mL/min/1.73 m^2^							
30 to <60	213	NE			NE		
≥60	256	Ref			Ref		
UACR, mg/gCr							
<30	301	Ref			Ref		
30 to <300	129	−0.11	−3.53, 3.31	0.947	−0.10	−2.13, 1.93	0.922
≥300	46	1.06	−4.26, 6.37	0.696	−0.71	−3.87, 2.44	0.655
NT-proBNP, pg/mL							
<125	371	Ref			Ref		
125 to <400	73	−1.64	−5.65, 2.37	0.421	−0.76	−3.14, 1.63	0.532
≥400	19	−0.77	−9.79, 8.26	0.867	−1.74	−7.10, 3.62	0.522
Plasma aldosterone concentration, pg/mL							
<120	426	Ref			Ref		
≥120	17	−4.77	−12.33, 2.80	0.216	−1.90	−6.39, 2.59	0.405
Plasma renin activity, ng/mL/h							
<1.0	183	Ref			Ref		
≥1.0	287	0.81	−2.24, 3.85	0.602	0.28	−1.53, 2.08	0.764
Diabetes							
No	200	Ref			Ref		
Yes	279	1.51	−2.36, 5.37	0.443	0.47	−1.82, 2.76	0.686
Dyslipidemia							
No	134	Ref			Ref		
Yes	285	−1.34	−4.61, 1.93	0.419	−0.15	−2.09, 1.79	0.881
Hyperuricemia							
No	325	Ref			Ref		
Yes	94	0.13	−3.46, 3.71	0.945	−0.26	−2.39, 1.87	0.811
Smoking habit							
No	396	Ref			Ref		
Yes	83	1.79	−2.51, 6.09	0.412	1.61	−0.94, 4.16	0.214
Drinking habit							
No	267	Ref			Ref		
Yes	209	1.17	−1.97, 4.31	0.462	−0.43	−2.29, 1.44	0.652
Initial dose of esaxerenone, mg							
1.25	230	Ref			Ref		
2.5	249	1.45	−1.88, 4.79	0.391	1.44	−0.54, 3.42	0.153

Estimates (95% CIs) indicate the absolute difference in BP change compared with the reference category. Negative estimates indicate a greater decrease in BP relative to the reference (i.e., a relatively stronger antihypertensive effect), whereas positive estimates indicate a smaller decrease in BP relative to the reference (i.e., a relatively weaker antihypertensive effect)

NE denotes variables excluded because the variance inflation factor was >5.0

*BMI* body mass index, *BP* blood pressure, *CCB* calcium channel blocker, *CI* confidence interval, *DBP* diastolic blood pressure, *eGFR*_*creat*_ creatinine-based estimated glomerular filtration rate, *FAS* full analysis set, *NE* not examined, *NT-proBNP* N-terminal pro-brain natriuretic peptide, *RAS* renin–angiotensin system, *Ref* reference, *SBP* systolic blood pressure, *UACR* urine albumin‐to‐creatinine ratio

Office SBP ≥ 140 mmHg (estimated change 2.38; *P* = 0.015) and CCB use (estimated change 3.47 mmHg; *P* = 0.002) were associated with a positive estimated change in bedtime home DBP from baseline to Week 12 after esaxerenone treatment.

Supplementary Table 2 shows results of linear regression analysis of office SBP and DBP changes from baseline to Week 12 in the FAS (secondary endpoint). Bedtime home SBP ≥ 135 (estimated change −3.49; *P* = 0.027) and initial esaxerenone dose of 2.5 mg (estimated change −3.35; *P* = 0.031) were associated with a negative estimated change in office DBP from baseline to Week 12.

### Background factors associated with a high incidence of elevated serum potassium levels

Background characteristics of the SAS by serum potassium level subgroups are summarized in Supplementary Table 3. Among the 492 patients in the SAS, 27 (5.5%) had a serum potassium level ≥5.5 mEq/L. Compared with patients with serum potassium level <5.5 mEq/L, those with serum potassium level ≥5.5 mEq/L tended to be older (66.1 vs 76.4 years), have higher baseline office SBP/DBP (142.5/82.1 vs 151.3/84.0 mmHg), longer duration of hypertension (9.3 vs 13.3 years), lower proportion of patients using a RAS inhibitor/CCB as a basal antihypertensive (25.2% vs 7.4%), lower eGFR_creat_ (66.2 vs 58.7 mL/min/1.73 m^2^) and UACR (112.1 vs 67.5 mg/gCr), higher NT-proBNP level (102.0 vs 180.7 pg/mL), and lower plasma aldosterone concentration (51.0 vs 39.1 pg/mL), respectively.

The results of logistic regression analysis for high incidence of serum potassium level ≥5.5 mEq/L in the SAS are shown in Table 5. Based on multivariate analysis, the only factor associated with a high incidence of serum potassium level ≥5.5 mEq/L was an elevated baseline serum potassium level (≥4.5 vs <4.5 mEq/L, OR 13.502, *P* < 0.001).

**Table 5:** Multivariate analysis for factors associated with serum potassium level ≥5.5 mEq/L in the SAS (n = 492)

	N	Serum potassium level ≥ 5.5 mEq/L			
		n	OR	95% CI	*P*
Age, years					
<65	177	3	Ref		
65 to <75	187	9	1.679	0.158, 17.861	0.667
≥75	128	15	4.800	0.441, 52.225	0.198
Office SBP, mmHg					
<140	210	7	Ref		
≥140	282	20	1.733	0.468, 6.414	0.410
Basal antihypertensive drugs					
RAS inhibitor	204	15	Ref		
CCB	169	10	0.885	0.219, 3.573	0.863
RAS inhibitor/CCB	119	2	0.253	0.035, 1.826	0.173
Serum potassium, mEq/L					
<4.5	361	7	Ref		
≥4.5	121	18	13.502	3.402, 53.583	<0.001
eGFR_creat_, mL/min/1.73 m^2^					
30 to <60	218	14	0.093	0.002, 3.799	0.209
≥60	264	11	Ref		
UACR, mg/gCr					
<30	309	18	Ref		
30 to <300	132	7	1.219	0.282, 5.272	0.791
≥300	48	2	0.261	0.021, 3.176	0.292
NT-proBNP, pg/mL					
<125	378	17	Ref		
125 to <400	78	6	0.840	0.220, 3.210	0.799
≥400	20	3	0.367	0.027, 4.957	0.451
Diabetes					
No	209	18	Ref		
Yes	283	9	0.321	0.075, 1.365	0.124
Initial dose of esaxerenone, mg					
1.25	236	13	Ref		
2.5	256	14	2.519	0.073, 86.370	0.609

Variables used in this analysis were age, eGFR_creat_, UACR, comorbidity (diabetes), and selected factors with *P* < 0.1 in the univariate analyses

*CCB* calcium channel blocker, *CI* confidence interval, *eGFR*_*creat*_ creatinine-based estimated glomerular filtration rate, *NT-proBNP* N-terminal pro-brain natriuretic peptide, *OR* odds ratio, *RAS* renin–angiotensin system, *Ref* reference, *SAS* safety analysis set, *SBP* systolic blood pressure, *UACR* urine albumin‐to‐creatinine ratio

## Discussion

This pooled analysis aimed to identify background factors associated with the home BP-lowering effect of esaxerenone as well as factors associated with a high incidence of elevated serum potassium levels in hypertensive patients with diverse underlying medical conditions in clinical settings. The factors that were associated with a strong BP-lowering effect of esaxerenone were female sex, the use of RAS inhibitors as a basal antihypertensive agent, and office pulse rate ≥100 beats/min. However, the 95% CI of office pulse rate ≥100 beats/min was wide (0.65, 49.54 mmHg), suggesting that the results depended on the performance of some patients. Patients with baseline serum potassium levels ≥4.5 mEq/L were shown to have an increased risk of developing elevated serum potassium levels (≥5.5 mEq/L) after treatment with esaxerenone. However, low eGFR and high UACR were significantly associated with a high incidence of elevated serum potassium levels in the previous pooled analysis but not in the present study.

The previous pooled analysis of seven phase 3 clinical trials identified several factors associated with the antihypertensive effect of esaxerenone, including female sex, low body weight, low plasma aldosterone concentration, short duration of hypertension, no history of antihypertensive medication, low UACR, and non-smoking status [29]. In the present study, female sex was the only consistent factor associated with the BP-lowering effect of esaxerenone. A new finding in this study was the stronger BP-lowering effect observed in patients using RAS inhibitors compared with CCBs as the basal antihypertensive.

This study had a small overall sample size and was exploratory in nature. The lack of identification of other factors associated with the BP-lowering effect of esaxerenone in the present study may be attributable to the overall smaller sample size of the present study (N = 479) compared with the pooled analysis of seven phase 3 clinical trials (N = 1466) [29]. Additionally, the trial patients included many treatment-naïve patients, whereas many of the patients in this study had a history of using basal antihypertensive drugs. In patients who have achieved some (if not sufficient) hypotensive effect with basal antihypertensive medications, it may be difficult to identify factors that influence the antihypertensive effects of esaxerenone.

Although the sample size was small in this study, the findings suggest that female sex may be associated with the BP-lowering effect of esaxerenone in real-world clinical practice. The female hormone “progesterone” has a high affinity for mineralocorticoid receptors and is considered to be an endogenous MRB. In elderly (postmenopausal) women, progesterone production decreases, and mineralocorticoid receptor activity increases, which may enhance the antihypertensive effect of esaxerenone. As this study did not assess pre- and post-menopausal progesterone levels, further investigation is needed to confirm whether progesterone enhances the antihypertensive effect of esaxerenone.

In this analysis, esaxerenone showed a stronger BP-lowering effect in patients taking RAS inhibitors as a basal antihypertensive drug compared with those taking CCBs. However, there were no significant differences in factors related to the antihypertensive effects of a RAS inhibitor, such as plasma renin activity. Among patients with hypertension undergoing long-term treatment with RAS inhibitors, approximately 30% to 50% experience an increase in the production of aldosterone as a compensatory mechanism, leading to higher levels of aldosterone in plasma; this phenomenon is also known as “aldosterone breakthrough” [32, 33]. Aldosterone breakthrough may have occurred in patients taking RAS inhibitors more than in patients taking CCBs. Therefore, this may have influenced the stronger antihypertensive effect of esaxerenone in patients taking RAS inhibitors than in patients taking CCBs.

In the previous pooled analysis, elevated baseline serum potassium levels were strongly associated with a high incidence of elevated serum potassium levels [29], which is consistent with the results of the present study. This indicates that baseline serum potassium level is a robust factor associated with increased serum potassium level in real-world clinical practice.

Some of the risk factors found to be associated with elevated serum potassium levels in previous clinical trials were not associated with elevated serum potassium levels in the current study. A previous analysis of phase 3 studies on esaxerenone identified patients with diabetes mellitus and albuminuria or proteinuria, patients with moderate renal dysfunction (eGFR 30–60 mL/min/1.73 m^2^), elderly patients, patients with high baseline serum potassium levels, and those receiving RAS inhibitors as being at risk for elevated serum potassium levels; these risks are described in the package insert [29–31]. These risk factors are generally consistent with those reported in previous studies of other MRBs, including eplerenone [34], finerenone [35], and spironolactone [36, 37]. Nevertheless, these were not extracted as risk factors in the present study. One of the reasons for this may be that this study was conducted using a different dosage regimen than that used in the clinical trials. That is, it is possible that dose reductions for high-risk patients and thorough monitoring in actual clinical conditions have contributed to the lower incidence of hyperkalemia observed in this study compared with clinical trials. The esaxerenone package insert specifies that patients with diabetes plus moderate renal dysfunction (eGFR 30–60 mL/min/1.73 m^2^) and albuminuria should receive half doses of esaxerenone [31]. In the five studies included in this pooled analysis, esaxerenone dosing was performed according to the package insert. The total number of cases with serum potassium levels ≥5.5 mEq/L was 27 (5.5%), which is considered a small number of cases to detect risk factors (7.9% in the previous pooled analysis [29]). Integration of a larger dataset is needed to improve the information and accuracy regarding risk factors associated with elevated serum potassium levels after esaxerenone treatment.

The present pooled analysis is subject to the limitations of each of the clinical studies from which the data were pooled (EX-DKD [21], EARLY-NH [23], ESES-LVH [24], EnaK [25], and EAGLE-DH [22]). The study had a small overall sample size and was exploratory in nature. Although female sex was found to be associated with the antihypertensive effect of esaxerenone in this study, pre- and post-menopausal progesterone levels were not investigated, and further investigation in a study with a larger sample size is needed to confirm whether this female hormone with high affinity for mineralocorticoid receptors enhances the antihypertensive effect of esaxerenone. The lack of identification of other factors associated with the BP-lowering effect of esaxerenone in the present study may be attributable to the small overall sample size. Additionally, differences in individual patient characteristics, such as medical history, overall health, response to medications (e.g., specific conditions or genetic factors), medication adherence, and other factors, may influence antihypertensive response. Although baseline serum potassium level ≥4.5 mEq/L was associated with an increased risk of developing serum potassium level ≥5.5 mEq/L in esaxerenone-treated patients, this cannot be conclusively determined as a new risk factor for elevated serum potassium levels in real-world clinical practice owing to the small sample size. In patients with serum potassium level ≥4.5 mEq/L, treatment with esaxerenone in addition to a RAS inhibitor should be carefully considered. The evaluation period of this study was 12 weeks, which may not have been sufficient to determine the safety of esaxerenone in terms of its effect on serum potassium levels and eGFR. In particular, patients with early diabetic kidney disease, who have a slower rate of eGFR decline, were not enrolled in this study, and the long-term safety in patients with early diabetic kidney disease remains unknown. Furthermore, although the study protocol excluded patients with secondary hypertension, including primary aldosteronism, it is possible that a certain number of patients with primary aldosteronism were included due to their not having a definitive diagnosis. Urinary biomarkers were calculated using a single spot urine collection, but not using multiple random urine collections on separate days or 24 h urine collections. This may have affected the analysis of correlations between urinary biomarkers and BP-lowering effects. The effect of dietary salt intake on the antihypertensive effect of esaxerenone was not investigated in this pooled analysis because data on salt intake were not available for all five studies. In addition, a position statement was recently published in which the use of spot urine samples for the estimation of an individual’s salt intake is strongly discouraged [38]; therefore, a cautious approach is required when estimating individuals’ salt intake. Finally, the study was conducted in Japan, and results may vary by geography and ethnic group.

In conclusion, esaxerenone provides a reliable BP-lowering effect in a broad group of patients with a wide range of background characteristics, but the factors that were associated with a strong BP-lowering effect of esaxerenone were female sex and the use of RAS inhibitors as a basal antihypertensive drug. Patients with baseline serum potassium levels ≥4.5 mEq/L were shown to have an increased risk of developing elevated serum potassium levels (≥5.5 mEq/L) after treatment with esaxerenone. Further research is needed to identify the optimal patient population for esaxerenone treatment.

## Supplementary information

Supplementary materials

## Data Availability

The anonymized data underlying the results presented in this manuscript may be made available to researchers upon submission of a reasonable request to the corresponding author. The decision to disclose the data will be made by the corresponding author and the funder, Daiichi Sankyo Co., Ltd. Data disclosure can be requested for 36 months from article publication.
