# Supplementary material for: Factors influencing the efficacy and safety of esaxerenone in hypertensive patients: a pooled analysis of five clinical studies on different comorbidities
Source: Hypertens Res. 2024 Aug 2;47(10):2826–39. doi: 10.1038/s41440-024-01818-0 (PMC11456503; doi:10.1038/s41440-024-01818-0)
Supplement: Supplementary file 1 — Supplementary materials [file 41440_2024_1818_MOESM1_ESM.docx]

**Supplementary materials**

**Supplementary Table 1.** Baseline demographic and clinical characteristics of patients in the five studies (FAS)

|  | **EX-DKD**  **N = 109** | **EARLY-NH**  **N = 93** | **ESES-LVH**  **N = 58** | **ENaK**  **N = 126** | **EAGLE-DH**  **N = 93** |
| --- | --- | --- | --- | --- | --- |
| Sex, n (%) |  |  |  |  |  |
| Male | 59 (54.1) | 47 (50.5) | 41 (70.7) | 66 (52.4) | 64 (68.8) |
| Female | 50 (45.9) | 46 (49.5) | 17 (29.3) | 60 (47.6) | 29 (31.2) |
| Age, years |  |  |  |  |  |
| n | 108 | 93 | 58 | 126 | 93 |
| Mean ± SD | 72.6 ± 7.0 | 67.6 ± 11.6 | 64.8 ± 12.7 | 61.2 ± 11.6 | 66.3 ± 9.9 |
| BMI, kg/m^2^ |  |  |  |  |  |
| n | 109 | 93 | 58 | 126 | 93 |
| Mean ± SD | 25.0 ± 3.8 | 25.5 ± 4.3 | 25.2 ± 4.1 | 25.9 ± 4.2 | 27.7 ± 4.1 |
| Complications, n (%) |  |  |  |  |  |
| Any | 107 (98.2)^a^ | 79 (84.9) | 52 (89.7) | 113 (89.7) | 89 (95.7)^a^ |
| Diabetes | 109 (100.0) | 33 (35.5) | 11 (19.0) | 33 (26.2) | 93 (100.0) |
| Dyslipidemia | 80 (73.4) | 52 (55.9) | 22 (37.9) | 74 (58.7) | 79 (84.9) |
| Hyperuricemia | 32 (29.4) | 17 (18.3) | 6 (10.3) | 18 (14.3) | 27 (29.0) |
| Morning home SBP, mmHg |  |  |  |  |  |
| n | 109 | 86 | 58 | 126 | 93 |
| Mean ± SD | 135.6 ± 12.1 | 143.8 ± 13.3 | 142.8 ± 8.1 | 136.7 ± 12.1 | 136.4 ± 10.8 |
| Morning home DBP, mmHg |  |  |  |  |  |
| n | 109 | 86 | 58 | 126 | 93 |
| Mean ± SD | 75.9 ± 9.3 | 86.7 ± 9.8 | 85.0 ± 10.0 | 88.0 ± 9.0 | 82.3 ± 9.6 |
| Bedtime home SBP, mmHg |  |  |  |  |  |
| n | 109 | 89 | 58 | 126 | 90 |
| Mean ± SD | 129.3 ± 13.8 | 135.1 ± 13.6 | 141.0 ± 8.4 | 130.5 ± 13.6 | 131.9 ± 12.2 |
| Bedtime home DBP, mmHg |  |  |  |  |  |
| n | 109 | 89 | 58 | 126 | 90 |
| Mean ± SD | 71.0 ± 10.0 | 80.5 ± 9.8 | 81.9 ± 10.5 | 82.0 ± 9.1 | 78.0 ± 11.1 |
| Office SBP, mmHg |  |  |  |  |  |
| n | 109 | 93 | 58 | 126 | 93 |
| Mean ± SD | 144.7 ± 10.8 | 147.2 ± 17.8 | 145.9 ± 14.3 | 141.4 ± 15.2 | 136.5 ± 15.5 |
| Office DBP, mmHg |  |  |  |  |  |
| n | 109 | 93 | 58 | 126 | 93 |
| Mean ± SD | 76.1 ± 9.6 | 84.1 ± 12.3 | 85.3 ± 12.8 | 86.5 ± 10.8 | 80.0 ± 10.4 |
| Morning home pulse rate, beats/min |  |  |  |  |  |
| n | 109 | 94 | - | 126 | 87 |
| Mean ± SD | 67.4 ± 9.2 | 66.2 ± 9.4 | - | 69.6 ± 10.0 | 69.0 ± 10.1 |
| Bedtime home pulse rate, beats/min |  |  |  |  |  |
| n | 109 | 96 | - | 126 | 84 |
| Mean ± SD | 70.3 ± 9.8 | 71.4 ± 10.6 | - | 73.9 ± 10.8 | 73.1 ± 10.5 |
| Office pulse rate, beats/min |  |  |  |  |  |
| n | 112 | 101 | 60 | 126 | 93 |
| Mean ± SD | 73.6 ± 12.1 | 74.3 ± 12.2 | 71.4 ± 11.2 | 73.4 ± 10.7 | 72.7 ± 10.9 |
| Duration of hypertension, years |  |  |  |  |  |
| n | 71 | 82 | 43 | 69 | 70 |
| Mean ± SD | 10.7 ± 7.9 | 140.2 ±122.1^b^ | 6.7 ± 8.4 | 6.0 ± 6.3 | 10.2 ± 7.5 |
| Basal antihypertensive, n (%) |  |  |  |  |  |
| RAS inhibitor | 36 (33.0) | 45 (48.4) | 19 (32.8) | 67 (53.2) | 31 (33.3) |
| CCB | 0 (0.0) | 48 (51.6) | 39 (67.2) | 59 (46.8) | 18 (19.4) |
| RAS inhibitor/CCB | 73 (67.0) | 0 (0.0) | 0 (0.0) | 0 (0.0) | 44 (47.3) |
| Serum potassium, mEq/L |  |  |  |  |  |
| n | 109 | 93 | 48 | 126 | 93 |
| Mean ± SD | 4.3 ± 0.3 | 4.0 ± 0.4 | 4.1 ± 0.5 | 4.3 ± 0.4 | 4.2 ± 0.4 |
| eGFR_creat_, mL/min/1.73 m^2^ |  |  |  |  |  |
| n | 109 | 93 | 48 | 126 | 93 |
| Mean ± SD | 49.4 ± 7.6 | 69.7 ± 17.1 | 71.3 ± 15.8 | 74.8 ± 14.9 | 66.8 ± 20.6 |
| UACR, mg/gCr |  |  |  |  |  |
| n | 109 | 93 | - | 124 | 93 |
| Mean ± SD | 184.0 ± 257.1 | 110.4 ± 321.5 | - | 34.6 ± 130.8 | 145.3 ± 631.9 |
| NT-proBNP, pg/mL |  |  |  |  |  |
| n | 105 | 93 | 58 | 117 | 90 |
| Mean ± SD | 128.6 ± 189.8 | 83.7 ± 92.3 | 191.8 ± 249.4 | 59.9 ± 61.5 | 92.2 ± 125.4 |
| Plasma aldosterone concentration, pg/mL |  |  |  |  |  |
| n | 105 | 93 | 58 | 94 | 93 |
| Mean ± SD | 75.2 ± 26.3 | 39.4 ± 36.8 | 37.6 ± 22.6 | 39.4 ± 26.8 | 46.4 ± 43.3 |
| Plasma renin activity, ng/mL/h |  |  |  |  |  |
| n | 105 | 93 | 58 | 121 | 93 |
| Mean ± SD | 3.2 ± 4.5 | 2.7 ± 5.8 | 1.3 ± 1.3 | 4.2 ± 12.8 | 5.4 ± 13.7 |
| Smoking habit, n (%) |  |  |  |  |  |
| No | 93 (85.3) | 79 (84.9) | 40 (69.0) | 109 (86.5) | 75 (80.6) |
| Yes | 16 (14.7) | 14 (15.1) | 18 (31.0) | 17 (13.5) | 18 (19.4) |
| Drinking habit, n (%) |  |  |  |  |  |
| No | 78 (71.6) | 51 (54.8) | 28 (48.3) | 57 (45.2) | 53 (57.0) |
| Yes | 29 (26.6) | 41 (44.1) | 30 (51.7) | 69 (54.8) | 40 (43.0) |
| Urinary sodium, mEq/L |  |  |  |  |  |
| n | 109 | 93 | 58 | 126 | 93 |
| Mean ± SD | 106.8 ± 43.0 | 113.5 ± 56.1 | 117.2 ± 63.4 | 108.0 ± 52.7 | 100.8 ± 41.4 |
| Urinary potassium, mEq/L |  |  |  |  |  |
| n | 109 | 93 | 57 | 126 | 93 |
| Mean ± SD | 38.4 ± 21.7 | 43.9 ± 29.7 | 37.3 ± 22.6 | 42.5 ± 23.5 | 38.2 ± 17.6 |
| Initial esaxerenone dose, n (%) |  |  |  |  |  |
| 1.25 mg | -^c^ | 38 (40.9) | 11 (19.0) | 24 (19.0) | 47 (50.5) |
| 2.5 mg | -^c^ | 55 (59.1) | 47 (81.0) | 102 (81.0) | 46 (49.5) |

^a^Not including diabetes.

^b^The unit of measure is months.

^c^The protocol specified 1.25 mg as the initial esaxerenone dose.

*BMI* body mass index, *CCB* calcium channel blocker, *DBP* diastolic blood pressure, *eGFR_creat_* creatinine-based estimated glomerular filtration rate, *FAS* full analysis set, *NT-proBNP* N-terminal pro-brain natriuretic peptide, *RAS* renin–angiotensin system, *SBP* systolic blood pressure, *SD* standard deviation, *UACR* urine albumin‐to‐creatinine ratio

**Supplementary Table 2.** Multivariate analysis of factors associated with a change from baseline to Week 12 in office SBP and DBP in the FAS (n = 479)

|  | **N** | **SBP** | | | | **DBP** | | | |
| --- | --- | --- | --- | --- | --- | --- | --- | --- | --- |
|  |  | **Estimated change**  **in BP, mmHg** | **95% CI** | | ***P*** | **Estimated change**  **in BP, mmHg** | **95% CI** | | ***P*** |
| Sex |  |  |  |  |  |  |  |  |  |
| Male | 277 | 4.12 | −1.09, 9.32 | | 0.121 | 1.39 | −1.73, 4.51 | | 0.382 |
| Female | 202 | Ref |  |  |  | Ref |  |  |  |
| Age, years |  |  |  |  |  |  |  |  |  |
| <65 | 175 | Ref |  |  |  | Ref |  |  |  |
| 65 to <75 | 185 | −1.82 | −7.31, 3.67 | | 0.514 | −2.39 | −5.68, 0.90 | | 0.154 |
| ≥75 | 119 | −4.99 | −11.89, 1.90 | | 0.155 | −3.34 | −7.47, 0.80 | | 0.113 |
| BMI, kg/m^2^ |  |  |  |  |  |  |  |  |  |
| <18.5 | 8 | 17.69 | −12.39, 47.77 | | 0.247 | −1.20 | −19.23, 16.83 | | 0.896 |
| 18.5 to <25 | 207 | Ref |  |  |  | Ref |  |  |  |
| ≥25 | 263 | −0.16 | −4.73, 4.42 | | 0.947 | 0.11 | −2.63, 2.85 | | 0.938 |
| Morning home SBP, mmHg |  |  |  |  |  |  |  |  |  |
| <135 | 187 | Ref |  |  |  | Ref |  |  |  |
| ≥135 | 285 | 1.82 | −3.51, 7.16 | | 0.502 | 2.90 | −0.30, 6.09 | | 0.076 |
| Morning home DBP, mmHg |  |  |  |  |  |  |  |  |  |
| <85 | 256 | Ref |  |  |  | Ref |  |  |  |
| ≥85 | 216 | 1.73 | −4.25, 7.72 | | 0.568 | 0.95 | −2.63, 4.54 | | 0.601 |
| Bedtime home SBP, mmHg |  |  |  |  |  |  |  |  |  |
| <135 | 268 | Ref |  |  |  | Ref |  |  |  |
| ≥135 | 204 | −2.43 | −7.57, 2.70 | | 0.351 | −3.49 | −6.57, −0.41 | | 0.027 |
| Bedtime home DBP, mmHg |  |  |  |  |  |  |  |  |  |
| <85 | 346 | Ref |  |  |  | Ref |  |  |  |
| ≥85 | 126 | −0.88 | −7.25, 5.48 | | 0.784 | −0.70 | −4.51, 3.12 | | 0.718 |
| Office SBP, mmHg |  |  |  |  |  |  |  |  |  |
| <140 | 204 | Ref |  |  |  | Ref |  |  |  |
| ≥140 | 275 | −8.86 | −13.72, −4.00 | | <0.001 | 1.01 | −1.91, 3.92 | | 0.497 |
| Office DBP, mmHg |  |  |  |  |  |  |  |  |  |
| <90 | 351 | Ref |  |  |  | Ref |  |  |  |
| ≥90 | 128 | −4.15 | −10.06, 1.76 | | 0.167 | −9.12 | −12.66, −5.58 | | <0.001 |
| Morning home pulse rate, beats/min |  |  |  |  |  |  |  |  |  |
| <60 | 73 | Ref |  |  |  | Ref |  |  |  |
| 60 to <100 | 384 | 4.49 | −4.59, 13.58 | | 0.331 | 0.75 | −4.70, 6.19 | | 0.786 |
| ≥100 | 2 | - |  |  |  | - |  |  |  |
| Bedtime home pulse rate, beats/min |  |  |  |  |  |  |  |  |  |
| <60 | 48 | Ref |  |  |  | Ref |  |  |  |
| 60 to <100 | 404 | 1.01 | −9.25, 11.26 | | 0.847 | 2.09 | −4.06, 8.24 | | 0.503 |
| ≥100 | 5 | - |  |  |  | - |  |  |  |
| Office pulse rate, beats/min |  |  |  |  |  |  |  |  |  |
| <60 | 35 | Ref |  |  |  | Ref |  |  |  |
| 60 to <100 | 435 | 2.59 | −8.07, 13.24 | | 0.633 | 0.36 | −6.02, 6.75 | | 0.911 |
| ≥100 | 9 | −4.48 | −23.47, 14.50 | | 0.642 | 0.87 | −10.51, 12.25 | | 0.880 |
| Duration of hypertension, years |  |  |  |  |  |  |  |  |  |
| <5 | 126 | Ref |  |  |  | Ref |  |  |  |
| 5 to <10 | 80 | −1.89 | −7.45, 3.67 | | 0.503 | −2.02 | −5.36, 1.31 | | 0.233 |
| ≥10 | 129 | 1.28 | −4.04, 6.59 | | 0.636 | −0.51 | −3.70, 2.67 | | 0.751 |
| Basal antihypertensive drugs |  |  |  |  |  |  |  |  |  |
| RAS inhibitor | 198 | Ref |  |  |  | Ref |  |  |  |
| CCB | 164 | 5.56 | −0.03, 11.16 | | 0.051 | 1.68 | −1.67, 5.03 | | 0.324 |
| RAS inhibitor/CCB | 117 | 0.58 | −5.00, 6.16 | | 0.839 | −0.86 | −4.20, 2.49 | | 0.614 |
| Serum potassium, mEq/L |  |  |  |  |  |  |  |  |  |
| <4.5 | 353 | Ref |  |  |  | Ref |  |  |  |
| ≥4.5 | 116 | 2.40 | −3.01, 7.81 | | 0.383 | 1.53 | −1.72, 4.77 | | 0.355 |
| eGFR_creat_, mL/min/1.73 m^2^ |  |  |  |  |  |  |  |  |  |
| 30 to <60 | 213 | NE |  |  |  | NE |  |  |  |
| ≥60 | 256 | Ref |  |  |  | Ref |  |  |  |
| UACR, mg/gCr |  |  |  |  |  |  |  |  |  |
| <30 | 301 | Ref |  |  |  | Ref |  |  |  |
| 30 to <300 | 129 | −2.89 | −8.12, 2.34 | | 0.278 | −2.27 | −5.40, 0.87 | | 0.156 |
| ≥300 | 46 | −4.34 | −12.64, 3.97 | | 0.304 | −2.07 | −7.04, 2.91 | | 0.413 |
| NT-proBNP, pg/mL |  |  |  |  |  |  |  |  |  |
| <125 | 371 | Ref |  |  |  | Ref |  |  |  |
| 125 to <400 | 73 | −0.12 | −6.25, 6.00 | | 0.968 | 1.65 | −2.02, 5.33 | | 0.375 |
| ≥400 | 19 | 8.77 | −4.25, 21.79 | | 0.186 | 0.14 | −7.67, 7.94 | | 0.973 |
| Plasma aldosterone concentration, pg/mL |  |  |  |  |  |  |  |  |  |
| <120 | 426 | Ref |  |  |  | Ref |  |  |  |
| ≥120 | 17 | −5.10 | −16.01, 5.80 | | 0.357 | −1.36 | −7.89, 5.18 | | 0.682 |
| Plasma renin activity, ng/mL/h |  |  |  |  |  |  |  |  |  |
| <1.0 | 183 | Ref |  |  |  | Ref |  |  |  |
| ≥1.0 | 287 | −0.97 | −5.61, 3.67 | | 0.680 | −1.26 | −4.04, 1.52 | | 0.374 |
| Complications |  |  |  |  |  |  |  |  |  |
| No | 36 | Ref |  |  |  | Ref |  |  |  |
| Yes | 440 | - |  |  |  | - |  |  |  |
| Diabetes |  |  |  |  |  |  |  |  |  |
| No | 200 | Ref |  |  |  | Ref |  |  |  |
| Yes | 279 | −1.13 | −6.97, 4.71 | | 0.703 | 0.89 | −2.61, 4.39 | | 0.617 |
| Dyslipidemia |  |  |  |  |  |  |  |  |  |
| No | 134 | Ref |  |  |  | Ref |  |  |  |
| Yes | 285 | 1.55 | −3.28, 6.37 | | 0.528 | −0.62 | −3.51, 2.27 | | 0.673 |
| Hyperuricemia |  |  |  |  |  |  |  |  |  |
| No | 325 | Ref |  |  |  | Ref |  |  |  |
| Yes | 94 | −1.66 | −7.19, 3.87 | | 0.554 | −1.33 | −4.65, 1.98 | | 0.429 |
| Smoking habit |  |  |  |  |  |  |  |  |  |
| No | 396 | Ref |  |  |  | Ref |  |  |  |
| Yes | 83 | −3.15 | −9.56, 3.26 | | 0.333 | −3.72 | −7.56, 0.12 | | 0.058 |
| Drinking habit |  |  |  |  |  |  |  |  |  |
| No | 267 | Ref |  |  |  | Ref |  |  |  |
| Yes | 209 | 3.24 | −1.55, 8.04 | | 0.184 | 2.47 | −0.40, 5.35 | | 0.091 |
| Initial dose of esaxerenone, mg |  |  |  |  |  |  |  |  |  |
| 1.25 | 230 | Ref |  |  |  | Ref |  |  |  |
| 2.5 | 249 | −4.70 | −9.78, 0.39 | | 0.070 | −3.35 | −6.40, −0.31 | | 0.031 |

Estimates (95% CIs) indicate the absolute difference in BP change compared with the reference category. Negative estimates indicate a greater decrease in BP relative to the reference (i.e., a relatively stronger antihypertensive effect), whereas positive estimates indicate a smaller decrease in BP relative to the reference (i.e., a relatively weaker antihypertensive effect).

NE denotes variables excluded because the variance inflation factor as >5.0.

*BMI* body mass index, *BP* blood pressure, *CCB* calcium channel blocker, *CI* confidence interval, *DBP* diastolic blood pressure, *eGFR_creat_* creatinine-based estimated glomerular filtration rate, *FAS* full analysis set, *NE* not examined, *NT-proBNP* N-terminal pro-brain natriuretic peptide, *RAS* renin–angiotensin system, *Ref* reference, *SBP* systolic blood pressure, *UACR* urine albumin‐to‐creatinine ratio

**Supplementary Table 3.** Background characteristics in the SAS by serum potassium level subgroups

|  | **SAS**  **N = 492** | **Serum potassium  <5.5 mEq/L**  **n = 465** | **Serum potassium  ≥5.5 mEq/L**  **n = 27** |
| --- | --- | --- | --- |
| Sex, n (%) |  |  |  |
| Male | 284 | 271 (58.3) | 13 (48.1) |
| Female | 208 | 194 (41.7) | 14 (51.9) |
| Age, years |  |  |  |
| N | 492 | 465 | 27 |
| Mean ± SD | 66.7 | 66.1 ± 11.2 | 76.4 ± 9.0 |
| BMI, kg/m^2^ |  |  |  |
| N | 490 | 463 | 27 |
| Mean ± SD | 25.9 | 25.9 ± 4.2 | 25.2 ± 3.6 |
| Morning home SBP, mmHg |  |  |  |
| N | 485 | 459 | 26 |
| Mean ± SD | 138.5 | 138.3 ± 12.0 | 142.2 ± 15.3 |
| Morning home DBP, mmHg |  |  |  |
| N | 485 | 459 | 26 |
| Mean ± SD | 83.4 | 83.4 ± 10.5 | 84.3 ± 11.1 |
| Bedtime home SBP, mmHg |  |  |  |
| N | 484 | 458 | 26 |
| Mean ± SD | 132.6 | 132.7 ± 13.3 | 130.4 ± 13.2 |
| Bedtime home DBP, mmHg |  |  |  |
| N | 484 | 458 | 26 |
| Mean ± SD | 78.4 | 78.5 ± 11.0 | 76.3 ± 8.7 |
| Office SBP, mmHg |  |  |  |
| N | 492 | 465 | 27 |
| Mean ± SD | 142.9 | 142.5 ± 15.3 | 151.3 ± 14.7 |
| Office DBP, mmHg |  |  |  |
| N | 492 | 465 | 27 |
| Mean ± SD | 82.2 | 82.1 ± 11.8 | 84.0 ± 10.1 |
| Morning home pulse rate, beats/min |  |  |  |
| N | 472 | 446 | 26 |
| Mean ± SD | 68.5 | 68.7 ± 9.8 | 64.3 ± 8.7 |
| Bedtime home pulse rate, beats/min |  |  |  |
| N | 469 | 443 | 26 |
| Mean ± SD | 72.5 | 72.6 ± 10.5 | 70.9 ± 8.8 |
| Office pulse rate, beats/min |  |  |  |
| N | 492 | 465 | 27 |
| Mean ± SD | 73.2 | 73.2 ± 11.3 | 73.1 ± 13.8 |
| Duration of hypertension, years |  |  |  |
| N | 347 | 327 | 20 |
| Mean ± SD | 9.5 | 9.3 ± 8.5 | 13.3 ± 11.8 |
| Basal antihypertensive, n (%) |  |  |  |
| RAS inhibitor | 204 | 189 (40.6) | 15 (55.6) |
| CCB | 169 | 159 (34.2) | 10 (37.0) |
| RAS inhibitor/CCB | 119 | 117 (25.2) | 2 (7.4) |
| Serum potassium, mEq/L |  |  |  |
| N | 482 | 457 | 25 |
| Mean ± SD | 4.20 | 4.2 ± 0.4 | 4.6 ± 0.3 |
| eGFR_creat_, mL/min/1.73 m^2^ |  |  |  |
| N | 482 | 457 | 25 |
| Mean ± SD | 65.8 | 66.2 ± 18.1 | 58.7 ± 14.2 |
| UACR, mg/gCr |  |  |  |
| N | 489 | 462 | 27 |
| Mean ± SD | 109.6 | 112.1 ± 354.2 | 67.5 ± 131.2 |
| NT-proBNP, pg/mL |  |  |  |
| N | 476 | 450 | 26 |
| Mean ± SD | 106.3 | 102.0 ± 150.6 | 180.7 ± 297.8 |
| Plasma aldosterone concentration, pg/mL |  |  |  |
| N | 456 | 431 | 25 |
| Mean ± SD | 50.3 | 51.0 ± 46.8 | 39.1 ± 40.2 |
| Plasma renin activity, ng/mL/h |  |  |  |
| N | 483 | 456 | 27 |
| Mean ± SD | 3.5 | 3.6 ± 9.7 | 2.0 ± 2.5 |
| Complications, n (%) | 452 | 427 (92.4) | 25 (92.6) |
| Diabetes | 283 | 274 (58.9) | 9 (33.3) |
| Dyslipidemia | 294 | 276 (68.0) | 18 (75.0) |
| Hyperuricemia | 95 | 90 (22.2) | 5 (20.8) |
| Smoking habit, n (%) |  |  |  |
| No | 408 | 383 (82.4) | 25 (92.6) |
| Yes | 84 | 82 (17.6) | 2 (7.4) |
| Drinking habit, n (%) |  |  |  |
| No | 275 | 258 (55.8) | 17 (63.0) |
| Yes | 214 | 204 (44.2) | 10 (37.0) |
| Urinary sodium, mEq/L |  |  |  |
| N | 492 | 465 | 27 |
| Mean ± SD | 108.5 | 107.9 ± 50.1 | 118.1 ± 58.7 |
| Urinary potassium, mEq/L |  |  |  |
| N | 491 | 464 | 27 |
| Mean ± SD | 40.4 | 40.3 ± 23.3 | 42.1 ± 24.9 |
| Initial esaxerenone dose, n (%) |  |  |  |
| 1.25 mg | 236 | 223 (48.0) | 13 (48.1) |
| 2.5 mg | 256 | 242 (52.0) | 14 (51.9) |

*BMI* body mass index, *CCB* calcium channel blocker, *DBP* diastolic blood pressure, *eGFR_creat_* creatinine-based estimated glomerular filtration rate, *FAS* full analysis set, *NT-proBNP* N-terminal pro-brain natriuretic peptide, *RAS* renin–angiotensin system, *SBP* systolic blood pressure, *SD* standard deviation, *UACR* urine albumin‐to‐creatinine ratio
